# Programmed Death (PD)-1-Deficient Mice Are Extremely Sensitive to Murine Hepatitis Virus Strain-3 (MHV-3) Infection

**DOI:** 10.1371/journal.ppat.1001347

**Published:** 2011-07-07

**Authors:** Yongwen Chen, Shengxi Wu, Guoning Guo, Lei Fei, Sheng Guo, Chengying Yang, Xiaolan Fu, Yuzhang Wu

**Affiliations:** 1 Institute of Immunology, PLA, Third Military Medical University, Chongqing, P. R. China; 2 Department of Chemistry and Bioengineering, Chongqing University of Technology, Chongqing, P. R. China; 3 Department of Emergency, SouthWest Hospital, PLA, Third Military Medical University, Chongqing, P. R. China; Washington University School of Medicine, United States of America

## Abstract

The inhibitory receptor programmed death-1 (PD-1) has the capacity to maintain peripheral tolerance and limit immunopathological damage; however, its precise role in fulminant viral hepatitis (FH) has yet to be described. Here, we investigated the functional mechanisms of PD-1 as related to FH pathogenesis induced by the murine hepatitis virus strain-3 (MHV-3). High levels of PD-1-positive CD4^+^, CD8^+^ T cells, NK cells and macrophages were observed in liver, spleen, lymph node and thymus tissues following MHV-3 infection. PD-1-deficient mice exhibited significantly higher expression of the effector molecule which initiates fibrinogen deposition, fibrinogen-like protein 2 (FGL2), than did their wild-type (WT) littermates. As a result, more severe tissue damage was produced and mortality rates were higher. Fluorescence double-staining revealed that FGL2 and PD-1 were not co-expressed on the same cells, while quantitative RT-PCR demonstrated that higher levels of IFN-γ and TNF-α mRNA transcription occurred in PD-1-deficient mice in response to MHV-3 infection. Conversely, *in vivo* blockade of IFN-γ and TNF-α led to efficient inhibition of FGL2 expression, greatly attenuated the development of tissue lesions, and ultimately reduced mortality. Thus, the up-regulation of FGL2 in PD-1-deficient mice was determined to be mediated by IFN-γ and TNF-α. Taken together, our results suggest that PD-1 signaling plays an essential role in decreasing the immunopathological damage induced by MHV-3 and that manipulation of this signal might be a useful strategy for FH immunotherapy.

## Author Summary

The principal characteristic of fulminant viral hepatitis (FH) induced by the murine hepatitis virus strain-3 (MHV-3) is severe hepatocellular necrosis, which is mediated by the fibrinogen-like protein 2 (FGL2), a molecule that has the capacity to promote fibrinogen deposition and activate the coagulation cascades. Here, we report that MHV-3 infection of program death-1 (PD-1)-deficient mice results in tissue damage throughout multiple organs, including the liver, spleen, thymus and lymph nodes. The liver damage, in particular, occurred earlier and was more severe in PD-1-deficient mice than in their wild type (WT) littermates. Further investigation determined that MHV-3 infection was associated with high levels of IFN-γ and TNF-α in the damaged organs of PD-1-deficient mice. Conversely, intraperitoneal injection of a combination of anti-IFN-γ and anti-TNF-α blocking mAbs led to inhibition of FGL2 expression, greatly attenuated tissue lesions and reduced mortality. Our results demonstrate that PD-1 signaling controls immunopathological damage following MHV-3 infection, indicating that manipulation of the PD-1 signal might represent a useful strategy for FH immunotherapy.

## Introduction

Although liver transplantation has emerged as an effective therapeutic approach for treating fulminant virus hepatitis (FH), mortality rates associated with FH remain very high worldwide [1]. The recent development of a mouse FH model, based upon infection with the murine hepatitis virus strain-3 (MHV-3), has provided insights into mechanisms underlying the disease pathogenesis and resulted in some novel treatment strategies [2].

MHV-3 is a single-stranded, positive-sense RNA virus that belongs to the Coronaviridae family. In inbred laboratory mice, the virus produces strain-dependent disease profiles that depend on the infection route, age, genetic background, and immune status of the host. For example, Balb/c, C57BL/6 and DBA/2 mice develop acute fulminant hepatitis, while C3H mice develop a mild chronic disease and mice of the A strain exhibit no evidence of hepatitis [3], [4]. In contrast to the resistant A strain mice, FH induced by MHV-3 in susceptible mice is characterized by the presence of sinusoidal thrombosis and hepatocellular necrosis [2], [3]. These pathological findings occur concomitantly with expression of fibrinogen-like protein 2 (FGL2), a virus-induced procoagulant molecule in the sinusoidal lining cells in the liver. FGL2 has the capacity to promote fibrinogen deposition and subsequently directly induce the coagulation cascades by the expression of procoagulant activity (PCA) [5]. Thus, up-regulation of FGL2 is an essential component of the lethal effects of MHV-3-induced FH.

Programmed death (PD)-1 is an inhibitory receptor expressed on activated T cells, B cells and myeloid cells. PD-1-deficient mice (*Pdcd1* ^−/−^) develop various spontaneous autoimmune diseases, including glomerulonephritis and dilated cardiomyopathy, indicating that this receptor plays a critical role in maintenance of peripheral tolerance [6]. PD-L1 (B7-H1) and PD-L2 (B7-DC), two immunoregulatory molecules belonging to the B7 superfamily, were identified as ligands for PD-1, engagement of PD-1 with its ligands mediates negative signaling events *via* recruitment of phosphatases, such as SHP-2, and dephosphorylation of some effector molecules involved in downstream T cell receptor (TCR) signaling [7], [8].

PD-1 signaling has also been shown to modulate the balance between antimicrobial immune defense and immune-mediated tissue damage. For example, PD-1-deficient mice develop more severe hepatocellular injury than their wild-type (WT) littermates in response to adenovirus infection [9]. In a herpes simplex virus (HSV) stromal keratitis mouse model, blockade of PD-1 signaling led to increased HSV-1-specific effector CD4^+^ T cell expansion, IFN-γ production, and exacerbated keratitis [10]. A functionally-significant high level of PD-1 expression has been found to be maintained by exhausted CD8^+^ T cells in mice chronically infected with lymphocytic choriomeningitis virus (LCMV), in primates exposed to simian immunodeficiency virus (SIV), and in humans suffering from infection with human immunodeficiency virus (HIV), hepatitis B or C virus (HBV or HCV), or human T-lymphotropic virus (HTLV). However, blockade of the PD-1/PD-Ls pathway efficiently restored the virus-specific effector functions of the exhausted T cells, and lead to substantially reduced in viral load [11], [12], [13], [14], [15]. The PD-1 signal is also known to play a key role in the chronicity of infections with bacteria (*Helicobacter pylori* and *Schistosoma mansoni*) [16], [17], pathogenic fungus (*Histoplasma capsulatum*) [18], and parasitic worms (*Taenia crassiceps*) [19]. It appears that a number of pathogenic microorganisms exploit the PD-1 signal in order to evade host immune responses and to establish persistent infection.

Although the influence of PD-1 signal activity has been studied in several infection models, there are no data available concerning the role of this pathway in FH. To this end, we used the MHV-3-induced mouse FH model to demonstrate that PD-1 signaling acts to limit the immunopathological damage during disease progression. Furthermore, our findings suggested that enhanced PD-1 signaling might represent a useful immunotherapeutic strategy for treating FH.

## Results

### PD-1 expression on immune cells increased in response to MHV-3 infection

**Figure 1:**
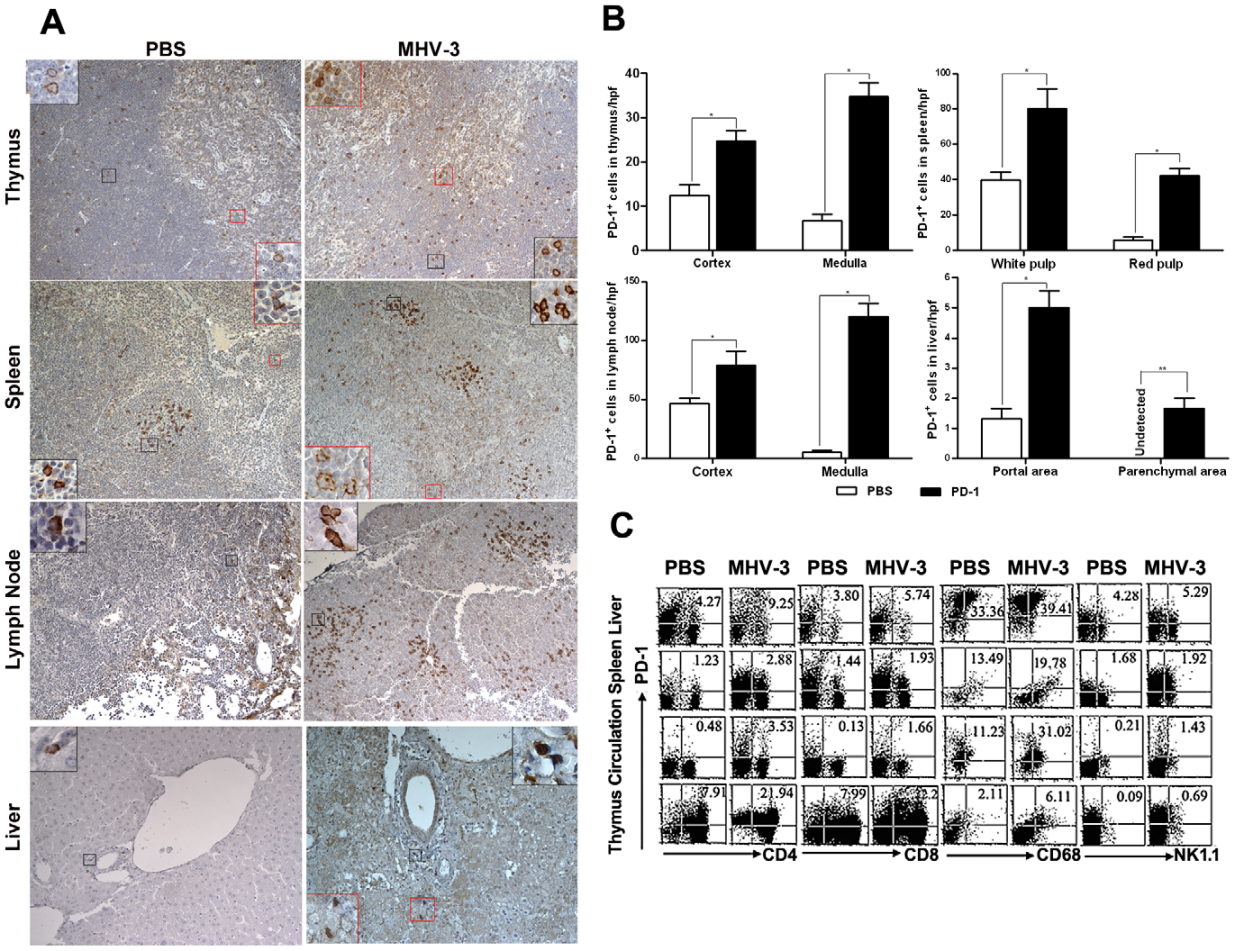
Enhanced expression of PD-1 on immune cells after 72 h of MHV-3 infection. (A) Immunohistochemical detection of PD-1 expression in mouse thymus, spleen, lymph nodes and liver. (B) Statistical analysis of the number of PD-1-positive ells in the indicated organs from MHV-3 infected mice or PBS-treated controls. (C) The expression of PD-1 on immune cells, including CD4^+^, CD8^+^ T cells, CD68^+^ macrophages and NK1.1^+^ NK cells in the indicated organs after 72 h of MHV-3 infection was measured by FACS. The number indicates the percentage of the positive cells in the indicated gate. One representative of three experiments which yielded similar results is shown. Magnification ×200. *p<0.05, **p<0.01. n = 8/group.

PD-1 expression has been previously described as being induced on specific cell subsets in response to viral or bacterial infection [20]. Thus, we first determined the status of PD-1 expression at 72 h after MHV-3 infection (10 PFU) by immunohistochemical techniques. PD-1-positive cells were observed in tissues from the thymus, spleen, lymph nodes and liver. Cellular expression was localized to the cell membrane and in the cytoplasma while was completely absent from the nuclear compartment. PD-1-positive cells were distributed throughout the medulla and cortex of the thymus and lymph nodes. In the spleen, PD-1-positive cells were restricted to the germinal center under normal conditions, but extended to the red pulp after infection. In infected liver, more PD-1-positive cells were present in the portal and parenchymal areas, as opposed to the relatively low presence of PD-1-positive cells in only the portal area in phosphate-buffered saline (PBS) treated-mice (Fig. 1A). The amount of PD-1-positive cells in the different organs of infected and control mice were counted and compared, results showed that the number of positive cells was significantly higher in infected mice (Fig. 1B). Furthermore, FACS analysis revealed that PD-1 expression was enhanced on multiple subsets of immune cells, including the CD4^+^ and CD8^+^ T cells, NK1.1^+^ NK cells and CD68^+^ macrophages (Fig. 1C). PD-1-positive cells were also observed in the lung, heart and kidney, however, the numbers of PD-1 positive cells in these tissues did not significantly increase in response to MHV-3 infection (Fig. S1).

### PD-1-deficient mice experienced multiple organ damage following MHV-3 infection

**Figure 2:**
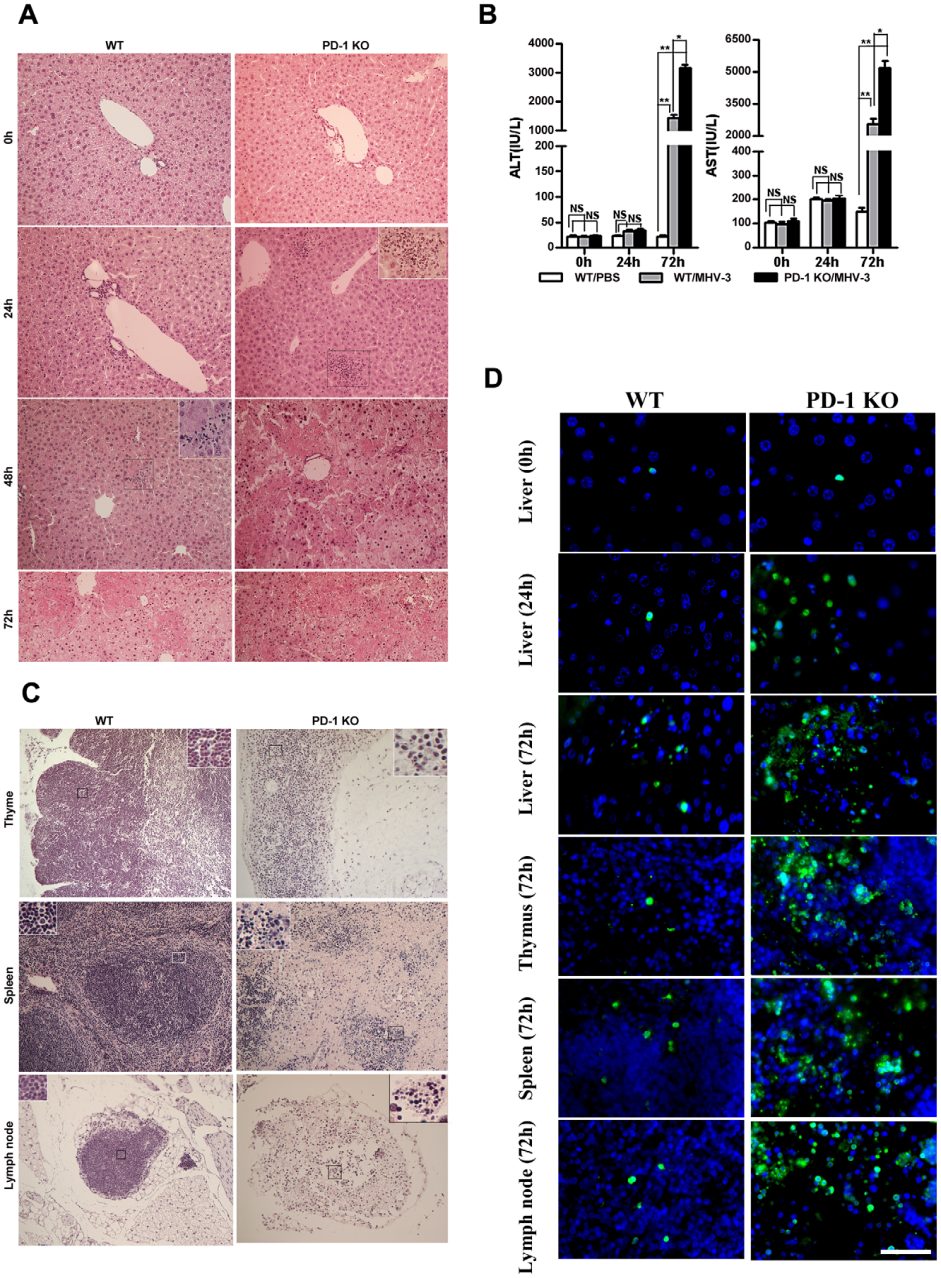
Multiple organ damage in PD-1-deficient mice after MHV-3 infection. (A) The architecture of liver from PD-1-deficient mice and WT littermates at different time points after MHV-3 infection was compared by H&E staining. (B) The ALT and AST levels between PD-1-deficient and WT mice were compared after MHV-3 infection. (C) The architecture of the thymus, spleen and lymph node from PD-1-deficient mice and WT littermates after 72 h of MHV-3 infection was compared by H&E staining. (D) Cellular apoptosis in the liver (0 h, 24 h and 72 h), thymus (72 h), spleen (72 h), and lymph nodes (72 h) from WT and PD-1-deficient mice after MHV-3 infection was analyzed by TUNEL staining. Blue color indicates nuclear DAPI staining. Scale bar  = 20 *µ*m. Magnification ×200. NS: not significantly different. *p<0.05 and **p<0.01. n = 4/group.

To investigate the potential role of PD-1 signaling in regulating FH tissue pathology, organs from MHV-3 infected PD-1-deficient (PD-1 KO) and WT mice were assessed for morphological differences. Small and discrete foci of necrosis with sparse polymorphonuclear leukocyte infiltration were observed in liver tissues from PD-1-deficient mice after 24 h of infection. In contrast, WT mice exhibited normal liver architecture at this time point. Slight liver damage became apparent in WT mice after 48 h of infection, meanwhile, the damaged areas of PD-1-deficient mice had enlarged and confluent necrosis had become evident. By 72 h of infection, the damaged region in PD-1-deficient mice had extended throughout the entire liver, while WT mice suffered much less damage and up to 60% of their liver tissue remained normal at this time point (Fig. 2A). Likewise, higher levels of alanine aminotransferase (ALT) and aspartate aminotransferase (AST) were observed in serum from PD-1-deficient mice after 72 h of infection (Fig. 2B). More interestingly, PD-1-deficient thymus, spleen and lymph node tissues infected with MHV-3 for 72 h exhibited severely disrupted architecture, loss of cellularity, and the presence of substantial amounts of karyorrhectic/apoptotic cell bodies. The histology of these organs from infected WT mice at 72 h was relatively normal (Fig. 2C). In conjunction with the apparent tissue necrosis, higher levels of cell apoptosis were also evidenced in the organs from PD-1-deficient mice by TUNEL staining (Fig. 2D). The architecture of other organs, including the heart, kidney and lung was relatively normal and only rare apoptosis events were observed in these tissues after infection (Fig. S2). In all, these results demonstrated that PD-1 deficiency led to enhanced pathological damage by MHV-3 in the liver, spleen, lymph node and thymus, where higher levels of PD-1-positive cells were found after infection.

### PD-1-deficient mice displayed higher mortality rates associated with MHV-3 infection

**Figure 3:**
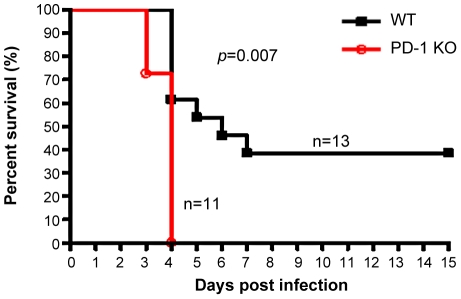
PD-1-deficiency resulted in higher mortality after MHV-3 infection. PD-1-deficient mice (n = 11) and WT littermates (n = 13) were infected with MHV-3 (10 PFU) and the survival rate was monitored for a total of 15 days. *p* = 0.007<0.05 was considered significantly different. One representative of four experiments that yielded similar results is shown.

The earlier and increased organ damage suffered by PD-1-deficient mice infected with MHV-3 instigated our monitoring of the mortality rates of PD-1-deficient mice and their similarly-infected (10 PFU) WT littermates. As shown in Fig. 3, all of the PD-1-deficient mice died within four days after infection, while 38% of the WT mice survived up to the end of the 15-day survey period (*p* = 0.007). These data indicated that PD-1 is likely a critical factor that controls MHV-3-mediated tissue damage and mortality.

### Elevated FGL2 expression was induced in PD-1-deficient mice following MHV-3 infection

**Figure 4:**
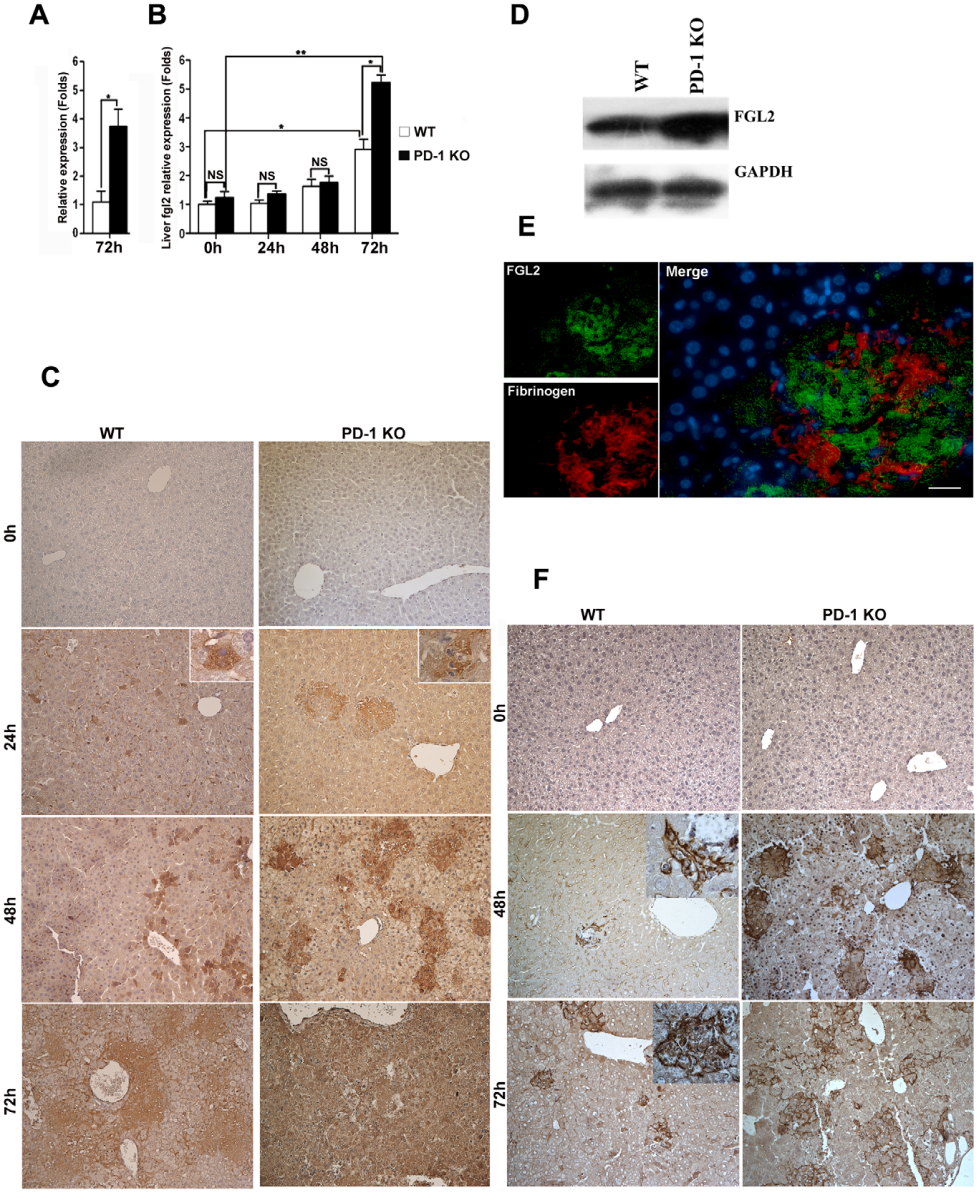
Higher FGL2 expression and stronger fibrinogen deposition in the liver of PD-1-deficient mice after MHV-3 infection. (A) The transcription levels of fgl2 in livers from PD-1-deficient and WT mice after 72 h of MHV-3 infection was detected by microarray analysis. (B) The transcription levels of fgl2 in livers from PD-1-deficient and WT mice after MHV-3 infection was detected by qPCR. (C) The expression of FGL2 in the livers of PD-1-deficient mice and WT littermates after MHV-3 infection was analyzed by immunohistochemistry. (D) The FGL2 protein level in liver tissues was detected by Western-blot (72 h of infection). (E) The expression of FGL2 and fibrinogen deposition in the liver was detected by fluorescent dual staining. (F) The deposition of fibrinogen in the liver of PD-1-deficient mice and WT controls at 0 h, 48 h and 72 h post MHV-3 infection was analyzed by immunohistochemistry. Blue color indicates nuclear DAPI staining. Scale bar  = 20 *µ*m. Magnification ×200. NS: not significant different. *p<0.05, ** *p*<0.01.

To understand the mechanisms of PD-1 deficiency-mediated tissue damage and mortality, we performed a comparative genome-wide microarray analysis (NimbleGen) of genes expressed in liver tissues of PD-1-deficient and WT mice after 72 h of MHV-3 infection. The most notable finding was pronounced up-regulation (3.75-fold) in the liver of PD-1-deficient mice of the fgl2 transcripts (Fig. 4A), the protein product of which has been demonstrated to induce lethality of MHV-3-induced FH [5]. In addition, the enhanced fgl2 expression was confirmed by quantitative (q)PCR, results revealed a 2.84-fold and 5.72-fold higher level was present in liver from WT and PD-1-deficient mice, respectively, after 72 h of MHV-3 infection, as compared to their uninfected controls. Moreover, its level in PD-1-deficient liver was 2.01-fold higher than that in the WT group at this time point (Fig. 4B). Immunohistochemistry was used to show that FGL2-positive cells were present in necrotic liver tissues in PD-1-deficient mice at 24 h after MHV-3 injection. The protein expression was found to be enhanced rapidly upon infection, and the highest level occurred at 72 h post-infection. However, occasional FGL2-positive cells were detected in the livers of WT mice at 24 h post-MHV-3 infection and these cells were also present, and slightly enhanced in number, at both the 48 h and 72 h time point (Fig. 4C). Western-blot was used to verify the higher FGL2 protein level in the livers of PD-1-deficient mice, as compared to WT littermates after 72 h of infection (Fig. 4D).

FGL2 has the capacity to induce fibrinogen deposition, which then activates the coagulation cascades and finally induces procoagulant activity. Therefore, the expression of FGL2 and fibrinogen deposition in damaged liver tissues was measured. Dual fluorescent staining evidenced that substantial fibrinogen deposition occurred in the FGL2-positive damaged liver tissue (Fig. 4E). Likewise, the level of fibrinogen deposition was more robust in livers from PD-1-deficient mice that in livers from WT littermates, at both the 48 h and 72 h time points (Fig. 4F).

**Figure 5:**
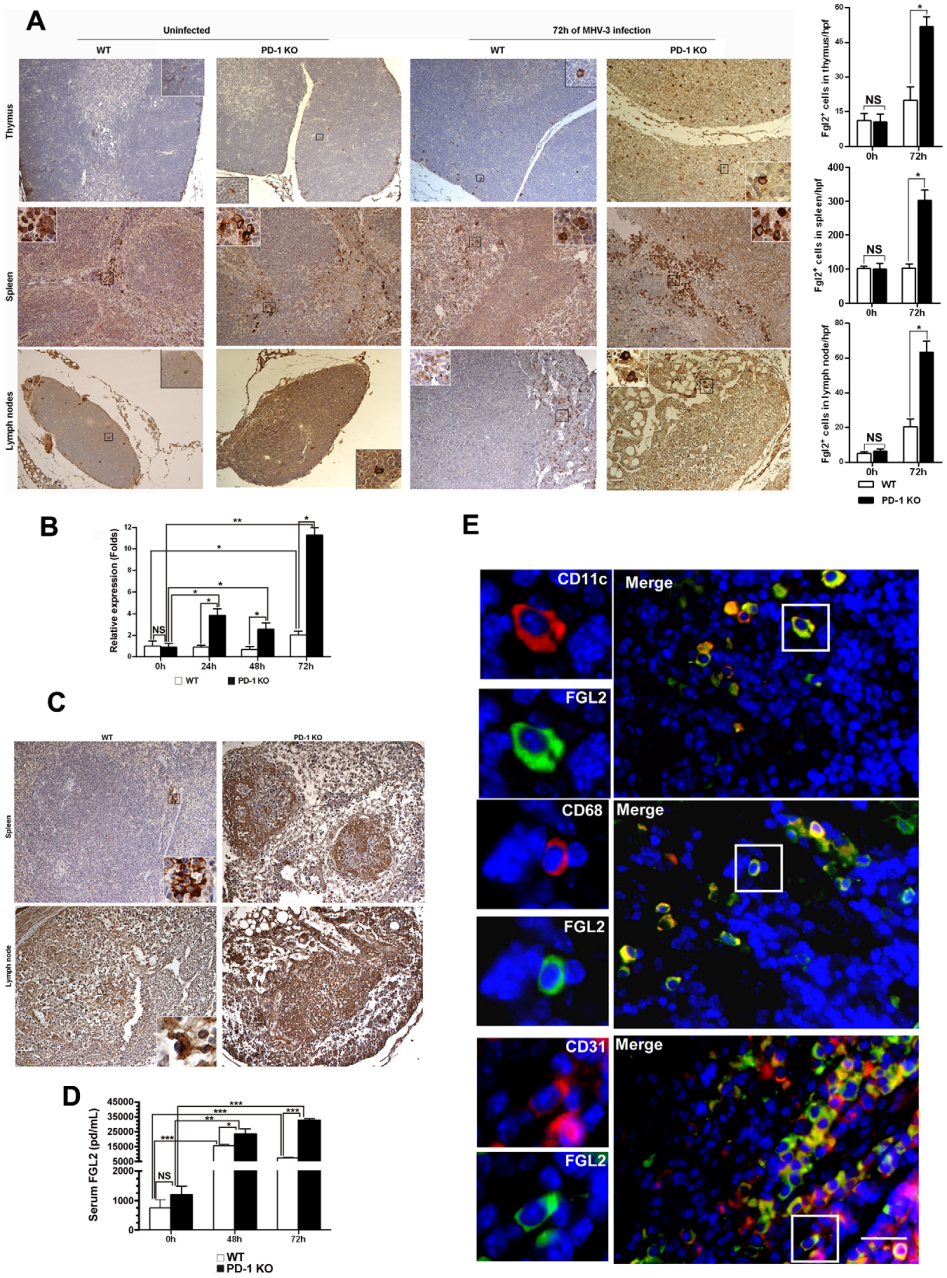
Enhanced FGL2 expression in the thymus, spleen, lymph nodes and serum of PD-1-deficient mice after MHV-3 infection. (A) The expression of FGL2 in the thymus, spleen and lymph nodes of PD-1-deficient mice and their WT littermates was detected by immunohistochemistry (left) and the amounts of FGL2-positive cells in these organs were counted and compared (right). (B) The fgl2 mRNA transcription in the spleens of PD-1-deficient mice and their WT littermates after MHV-3 infection was measured by qPCR. (C) The fibrinogen deposition in the spleen and lymph nodes was detected by immunohistochemistry. (D) The differential serum FGL2 level between PD-1 deficient and WT mice infected with MHV-3 was measured by ELISA. (E) The source of FGL2 was analyzed by fluorescent dual staining. Blue color indicates nuclear DAPI staining. Scale bar  = 20 *µ*m. Magnification ×200. NS: not significantly different. *p<0.05, **p<0.01 and ***p<0.001. n = 5/group.

To determine whether FGL2-mediated PCA activity was also involved in inducing damage in the other organs of PD-1-deficient mice, the expression of FGL2 was analyzed in the thymus, spleen and lymph nodes. Immunohistochemistry evidenced that FGL2-positive cells were also present in these organs. In thymus and lymph nodes, FGL2-positive cells were detected in both the medulla and cortex. In spleen, however, the positive cells were only found in the red pulp. Again, the expression of FGL2 appeared to be restricted to the cell membrane and cytoplasma. The distribution of FGL2-positive cells in PD-1-deficient mice had not changed after 72 h of MHV-3 infection, but the number of positive cells in the examined organs was enhanced significantly and the levels of expression were much stronger (Fig. 5A). In addition, the transcription of fgl2 in the spleen of PD-1-deficient mice was also significantly increased in response to infection (Fig. 5B). Meanwhile, higher levels of fibrinogen deposition were found in the spleen and lymph node tissues of PD-1-deficient mice (Fig. 5C). Moreover, the level of FGL2 present in serum, as measured by ELISA, was found to increase rapidly after infection, and the level in PD-1-deficient mice was significantly higher than that in WT littermates (Fig. 5D). To clarify the source of FGL2, fluorescent dual staining was performed on spleen tissues and results demonstrated that FGL2 was principally associated with CD11c-positive dendritic cells (DCs), CD68-positive macrophages and CD31-positive endothelial cells (Fig. 5E). All of these results indicated that the absence of PD-1 signaling can result in enhanced FGL2 expression, consequently inducing stronger fibrinogen deposition and more severe tissue necrosis in PD-1-deficient mice following MHV-3 infection.

### FGL2 expression is regulated by IFN-γ and TNF-α

**Figure 6:**
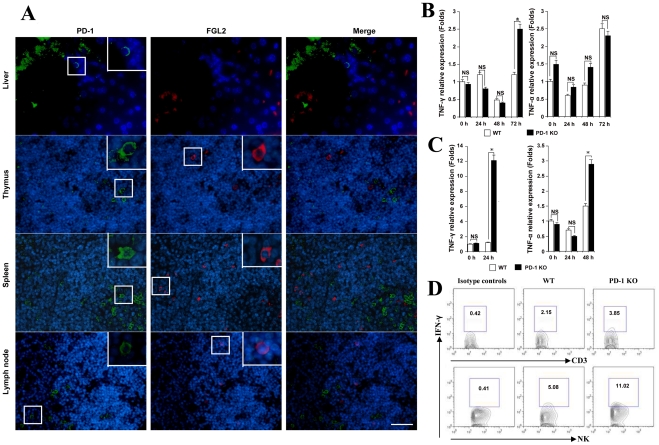
MHV-3 infection induced high levels of IFN-γ and TNF-α in PD-1-deficient mice. (A) The expression of FGL2 and PD-1 in liver, thymus, spleen and lymph nodes was detected by fluorescent dual staining. (B) Levels of IFN-γ and TNF-α mRNA in the liver and (C) in the spleen from PD-1-deficient mice and their WT littermates after MHV-3 infection was detected by qPCR. (D) The secretion of IFN-γ from NK cells and CD3^+^T cells in the liver from PD-1-deficient mice and their WT littermates after 72 h of MHV-3 infection was measured by FACs. The number indicates the percentage of the positive cells in the indicated gate. One representative of three experiments that yielded similar results is shown. Blue color indicates nuclear DAPI staining. Scale bar  = 20 *µ*m. NS: not significantly different. *p<0.05.

We further examined whether FGL2 secretion was regulated by PD-1 directly or indirectly. FGL2/PD-1 dual fluorescent staining was performed and results indicated that FGL2 and PD-1 were not co-expressed on the same cells in the liver, thymus, spleen or lymph nodes (Fig. 6A).

Previous studies have shown that the secretion of FGL2 can be triggered by the pro-inflammatory factors IFN-γ and TNF-α [21], [22]. On the other hand, the production of IFN-γ and TNF-α by activated T cells, NK cells and macrophages can be inhibited by the PD-1 signal [6]. Therefore, we compared the status of IFN-γ and TNF-α in PD-1-deficient and WT mice in response to MHV-3 infection. qPCR revealed that the transcription of the IFN-γ gene in liver was significantly higher in PD-1-deficient mice than in WT mice at 72 h post-MHV-3 infection (Fig. 6B). In PD-1-deficient spleen, the transcription of both IFN-γ and TNF-α was found to be rapidly enhanced upon MHV-3 exposure (Fig. 6C). FACs analysis indicated that IFN-γ secretion from NK cells, but not from CD3^+^T cells, in the liver was much higher in PD-1-deficient mice at 72 h after MHV-3 infection (Fig. 6D). The fact that IFN-γ and TNF-α both have the capacity to initiate FGL2 expression may explain why higher FGL2 expression was observed in the PD-1-deficient mice.

**Figure 7:**
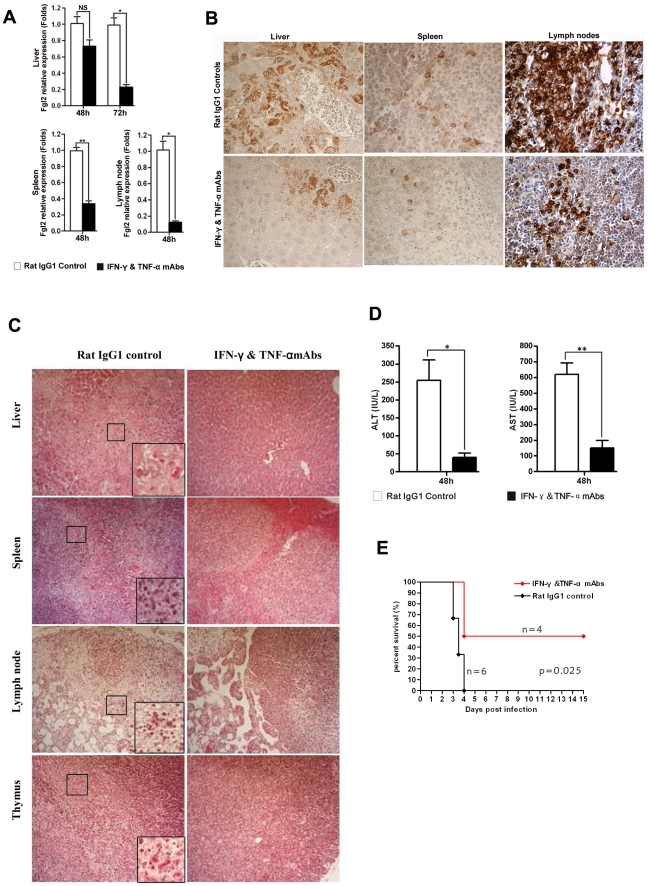
Up-regulation of FGL2 in PD-1-deficient mice after MHV-3 infection is regulated by IFN-γ and TNF-α. (A) Fgl2 mRNA and (B) its protein expression in the liver, spleen and lymph node from PD-1-deficient mice after 72 h of MHV-3 infection in the presence of IFN-γ and TNF-α mAbs or rat IgG1 isotype control antibodies was detected by qPCR and immunohistochemistry, respectively. (C) The IFN-γ and TNF-α mAbs treatment resulted in decreased damage to the liver, spleen, lymph node and thymus after 72 h of MHV-3 infection. (D) Reduced FGL2 level by IFN-γ and TNF-α mAbs treatment resulted in reduced liver damage (indicated by AST and ALT levels). (E) PD-1-deficient mice were infected with MHV-3 (10 PFU) and simultaneously treated with IFN-γ and TNF-α blocking mAbs (n = 4) or rat IgG1 control (n = 6), the survival rate was monitored for a total of 15 days. *p* = 0.025<0.05 was considered significantly different. One representative of three experiments that yielded similar results is shown. Magnification × 400. NS: not significantly different. *p<0.05 and **p<0.01. n = 5/group.

To further demonstrate that IFN-γ and TNF-α were responsible for the observed FGL2 up-regulation in MHV-3 infected PD-1-deficient mice, PD-1-deficient mice were infected with MHV-3 and simultaneously treated with a combination injection of anti-IFN-γ and anti-TNF-α blocking mAbs. The expression of fgl2 was measured by qPCR and protein detected by immunohistochemistry. The transcription of fgl2 mRNA (Fig. 7A) and its protein levels (Fig. 7B) were completely inhibited by 48 h after injection of anti-IFN-γ and anti-TNF-α mAbs, as compared to the control rat IgG1 isotype antibodies-treated group. Moreover, the tissue necrosis (Fig. 7C) and liver damage (as indicated by ALT and AST levels) (Fig. 7D) in PD-1-deficient mice were also significantly reduced, thus the MHV-3-mediated mortality rates were decreased as well (Fig. 7E).

## Discussion

The PD-1 signaling is best known for its ability to inhibit or dampen the immune response. Most of the evidence for this function, however, comes from models of tolerance or chronic infections [11, 12, 13, 14 15]. Although some studies have indicated that this signal might also participate in regulating acute infections [23], [24], [25], [26], its functions in this disease condition are much less clear. Here, we used a mouse FH model mediated by MHV-3 infection to describe the effects of PD-1 in this disease process. Firstly, PD-1 was found to be significantly up-regulated on T cells, macrophages and NK cells within the thymus, spleen, lymph nodes and liver in response to MHV-3 infection. To determine the exact role of PD-1 in the pathogenesis of FH, PD-1-deficient mice were used to establish an infection model. Interestingly, MHV-3-induced liver damage in PD-1-deficient mice occurred rapidly and the lesion area was much larger than in their WT littermates. We then extended our investigation to the thymus, spleen and lymph nodes, where increased PD-1-positive cells were observed post-infection. Surprisingly, severe tissue necrosis and substantial apoptosis was observed in these organs of PD-1-deficient mice at 72 h after MHV-3 infection. In contrast, these organs from WT mice exhibited relatively normal histology, a finding in agreement with previously reported results [27]. Taken together, these results suggested that PD-1 deficiency promoted expansion of the pathological damage from the liver to the lymph organs, including the spleen, lymph node and thymus in this FH model, thereafter, the absence of PD-1 was associated with higher mortality rates in response to MHV-3 infection.

Murine FH induced by MHV-3 is a recognized and validated model for studying host resistance/susceptibility to human hepatitis virus, and several studies have shown that BALB/c or C57BL/6 mice have an innate susceptibility to the infection [3], [4]. FGL2 has been proposed as a critical mediating factor of lethality in the MHV-3-induced FH mice due to the fact that it has the capacity to induce fibrinogen deposition, which in turn activates the coagulation cascades and induces procoagulant activity [5]. To clarify whether the tissue necrosis we observed in PD-1-deficient mice following infection was also mediated by FGL2, the expression of FGL2 was analyzed. Results showed that the expression of FGL2 was principally associated with CD31-positive endothelial cells, CD68-positive macrophages and CD11c-positive DCs. Surprisingly, significantly higher levels of FGL2 were observed after infection in all of PD-1-deficient organs, including the liver, thymus, spleen, lymph nodes, and serum than that in those from WT littermates. In addition, increased fibrinogen deposition was observed in the organs of PD-1-deficient mice. Although we currently have no direct data to evidence that FGL2 directly mediates the mortality of our PD-1-deficient mice, data from other researchers have clearly shown that FGL2 promoted mouse mortality in response to MHV-3 infection [5], [28], [29], [30]. Considering this, our results strongly indicate that the mortality of PD-1-deficient mice post-MHV-3 infection is due to the higher level of FGL2 secretion and increased fibrinogen deposition.

Indeed, it has been reported that both FGL2 and PD-1 are expressed on T cells, macrophages, and DCs, and that targeted deletion of fgl2 or PD-1 leads to impaired T cell activity, and these events are related to the development of autoimmune diseases [6], [31], [32]. We here also observed PD-1 expression as being enhanced on T cells (both CD4^+^ and CD8^+^ T cells). It was reasonable to propose that the expression of FGL2 may have been directly regulated by PD-1 signals. Unexpectedly, our FGL2/PD-1 dual staining showed that PD-1-positive cells in the liver, thymus, spleen and lymph nodes did not co-express FGL2, indicating that the expression of FGL2 was not directly regulated by PD-1. On the other hand, the expression of FGL2 is believed to be induced by IFN-γ and TNF-α [21], [22], while PD-1 signaling has the capacity to inhibit IFN-γ and TNF-α secretion from PD-1-positive immune cells [6]. Therefore, we evaluated and compared the status of IFN-γ and TNF-α in both PD-1-deficient and WT mice. Definitively, the transcription of IFN-γ and TNF-α genes was rapidly enhanced post-MHV-3 infection in PD-1-deficient mice, as compared to WT controls. In particular, a higher level of IFN-γ was observed in NK cells but not in CD3^+^ T cells of PD-1-deficient liver post-MHV-3 infection, indicating that the PD-1 signal can inhibit IFN-γ secretion from NK cells under such condition. Conversely, injection of a the combination of anti-IFN-γ and anti-TNF-α blocking mAbs was able to successfully inhibit fgl2 mRNA transcription and protein expression, resulting in reduced tissue damage and significantly protecting against MHV-3-mediated mortality in these mice. These results demonstrated that up-regulation of FGL2 in PD-1-deficient mice after MHV-3 infection was controlled, at least partially, by IFN-γ and TNF-α.

Recently, the secretion of FGL2 from naturally occurring CD4^+^Foxp3^+^ regulatory T cells (Tregs) was demonstrated and it was reported that deficiency of Treg-produced FGL2 resulted in increased effector T cell proliferation [32]. More interestingly, Levy and colleagues showed that the frequency of FGL2^+^ Tregs was higher in lymphoid tissues of MHV-3 infected mice, and treatment with FGL2-specific antibodies reversed MHV-3-induced liver injury and mortality *in vivo*. These findings demonstrated that FGL2 is an important effector cytokine of Tregs that contributes to MHV-3-induced FH [30]. PD-1 signaling has also been described as participating in regulation of Treg differentiation and function [33], [34]. In our study, we also analyzed the status of Foxp3^+^ cells in both PD-1-deficient and WT controls. However, the number of Foxp3-positive cells in the liver, spleen, lymph node or thymus was not significantly different between PD-1-deficient mice and their WT littermates after 72 h of MHV-3 infection (Fig. S3). Therefore, Foxp3^+^ cells are unlikely to be involved in the mortality of PD-1-deficient mice. However, the functional status of these Tregs (for example, the level of FGL2 secretion) in PD-1-deficienct mice requires further investigation, and such studies are in progress in our lab.

In conclusion, we have determined that PD-1 signaling can limit the immunopathological damage induced by MHV-3 infection in a mouse FH model. Our results suggest that enhancing the PD-1 signal by an immunotherapeutic approach might be a useful treatment for FH.

## Materials and Methods

### Ethics statement

All experiments were approved by and conducted in accordance with the guidelines of the Animal Care and Use Committee of the Third Military Medical University. All efforts were made to minimize animals' suffering.

### Mice

PD-1-KO-N10 (strain: BALB/cJ) mice were kindly provided by Prof. T. Honjo (Department of Immunology and Genomic Medicine, Kyoto University, Japan). The WT control mice were purchased from the Animal Center of Beijing University School of Medicine. All mice were maintained in micro-isolator cages and housed in the animal colony at the Animal Center, Third Military Medical University, standard laboratory chow diet and water was supplied *ad libitum*. Mice were used in experimental analysis at age of six weeks and at an average weight of 17 g (range: 16∼18 g).

### Virus and infection

MHV-3 was kindly provided by Prof. Q. Ning (Institute of Infectious Disease, Tongji Hospital of Tongji Medical College, Wuhan, China). The virus was plaque-purified and then expanded in murine L2 cells. Virus-containing supernatants were collected and stored at -80°C until use. Mice were intraperitoneally (i.p.) injected with 10 PFU/mouse in a total volume of 200 µl. In some experiments, PD-1-deficient mice were infected with MHV-3 (10 PFU) and simultaneously treated with a combination injection of anti-IFN-γ (200 µg/mouse per day, clone: R4-6A2, eBioscience, San Diego, CA, USA) and anti-TNF-α (200 µg/mouse per day, clone: MP6-XT22, eBioscience) mAbs, tissues were isolated for hematoxylin and eosin (H&E) staining to detect damage, and for fgl2 mRNA transcription measured by qPCR (see below). Serum ALT and AST levels were measured by an AU5400 automatic biochemistryanalyzer (OLYMPUS, Japan). In order to monitor the mortality, anti-IFN-γ and anti-TNF-α blocking mAbs or rat IgG1 control mAbs were injected everyday for a total of 6 days.

### Immunohistochemical and immunofluorescence staining

Paraffin-embedded tissue blocks were cut into 5 µm slices which were mounted on polylysine-charged glass slides. Endogenous peroxidase activity was blocked by exposure to 3.0% H_2_O_2_ for 15 min. Antigen retrieval was performed in a citrate buffer (pH 6.0) at 120°C for 10 min. Sections were then incubated at 4°C overnight with anti-mouse FGL2 (1∶100, mouse IgG, Santa Cruz, CA, USA), PD-1 (5 *µ*g/ml, goat IgG, R&D Systems, Minneapolis, MN, USA), CD11c (1∶50, rabbit IgG, Santa Cruz), CD68 (1∶50, rat IgG, eBioscience), CD31 (1∶100, rabbit IgG, Santa Cruz) or fibrinogen (1∶100, mouse IgG1, Dako, Capenteria, CA, USA). Immunoreactivity was detected by using a fluorescein isothiocyanate (FITC)-conjugated (1∶100, Zymed, San Francisco, CA, USA) or Cy3-conjugated secondary antibodies (1∶200; Jackson ImmunoResearch, West Grove, PA, USA). Results were analyzed by fluorescence microscopy (Axioplan 2, Zeiss, Germany). For immunohistochemical staining, HRP-conjugated anti-mouse, anti-goat or anti-rabbit IgG (1∶200, Zymed) was used, and the results were visualized with diaminobenzidine (DAB, Dako). Sections incubated with secondary antibodies only were used as isotype controls.

PD-1-, FGL2- or Foxp3-positive cells were determined by image analysis of histological sections. Photomicrographs were obtained in high-power fields (hpf, 0.625 mm^2^) and captured for analysis using Image Pro-Plus 5.0 software (Media Cybernetics, Silver Spring, MD, USA). The distribution of FGL2 and Foxp3 in thymus and lymph node is restricted in medulla and only the positive cells in this area were calculated. The number of PD-1-, FGL2- or Foxp3-positive cells per hpf were counted and expressed as the mean ± standard error of the mean (SEM). Moreover, analysis of tissue damage was based on H&E staining.

### Flow cytometry

The expression of PD-1 on immune cells (CD4, CD8, NK and macrophages) from different organs was assessed by flow cytometry (FACsAria Cytometer; Becton Dickinson, Germany). Briefly, cell suspensions of liver, spleen, blood and thymus tissues were washed and resuspended in PBS. Cells were then incubated for 30 min at room-temperature in the dark using primary antibodies (PE-PD-1, FITC-CD4, FITC-CD8, FITC-NK1.1 and FITC-CD68. eBioscience). To analyze the source of IFN-γ in the liver, PD-1-deficient and WT mice were treated with MHV-3 (10 PFU). After 72 h, liver tissues were isolated and mechanically homogenized, lymphocytes were collected thereafter. Cells were then treated with Brefeldin A solution (BFA) for 4 h, and FITC-NK1.1, FITC-CD3 or PE-IFN-γ mAbs (eBioscience) were added and the solution incubated for an additional 1 h. For each analysis, 10000 cells were evaluated. Flow cytometric data were analyzed with CellQuest Pro Software.

### Microarray analysis

The microarray experiment was performed under contact by Kangcheng Co. Ltd. (Shanghai, China). Briefly, total RNA was isolated by Trizol from liver tissue of PD-1-deficient and WT mice treated with 10 PFU MHV-3 for 72 h. RNA concentration was measured on the ND-1000 spectrophotometer (Nanodrop, Wilmington, DE, USA) and quality evaluated by denaturing gel electrophoresis. Samples were then amplified and labeled using a NimbleGen One-Color DNA Labeling Kit and hybridized using the NimbleGen Hybridization System (Roche Applied Science, Shanghai, China). After hybridization and washing, the processed slides were scanned by the Axon GenePix 4000B microarray scanner. Three independent experiments were performed, and for each test and control sample, two hybridizations were carried out by a reverse fluorescent strategy. Only genes whose alteration tendency was concordant between both microarray assays were selected as differentially expressed genes.

### Quantitative RT-PCR

Total RNA from the liver and spleen of WT and PD-1-deficient mice was isolated by Trizol (Invitrogen, Carlsbad, CA, USA), according to the manufacturer's instructions. RNA samples were quantitated by measurement of optical density at 260 nm. Total mRNA (2 µg) was reverse-transcribed to cDNA using the RevertAid H Minus First Strand cDNA Synthesis Kit (Fermentas China, Shenzhen City, China), in accordance with the manufacturer's instructions. qPCR was performed to quantitatively analyze the gene transcription levels of fgl2, IFN-γ and TNF-α genes. The primers for fgl2 were: sense 5′-TGGACAACAAAGTGGCAAATCT-3′ and anti-sense 5′-TGGAACACTTGCCATCCAAA-3′. The primers for IFN-γ were: sense 5′-TCAAGTGGCATAGATGTGGAAG-3′, and anti-sense 5′-CGCTTATGTTGTTGCTGATGG-3′. The primers for TNF-α were: sense 5′-CACGCTCTTCTGTCTACTGAAC-3′ and anti-sense 5′-ATCTGAGTGTGAGGGTCTGG-3′. The primers for β-actin (internal control) were: sense 5′-CACTATCGGCAATGAGCGGTTCC-3′ and anti-sense 5′-CAGCACTGTGTTGGCA TAGAGGTC-3′. The qPCR was performed at 95°C for 10 s followed by 40 cycles of 95°C for 5 s, 60°C for 15 s, and 72°C for 15 s. The specificity of PCR product was examined by a dissociation curve, and results were analyzed by the 2^−ΔΔCT^ method [35].

### Western-blot

The expression of FGL2 in liver from MHV-3 infected (72 h) PD-1-deficient mice or their WT littermates was determined by Western-blot; the protocol has been described previously [36].

### Enzyme-linked immunosorbent assay (ELISA)

The serum FGL2 level from mice infected with or without MHV-3 was detected by using the mouse FGL2 ELISA Kit (Cat: E90512Mu; Uscn Life Science Inc., Wuhan, China) and following the manufacturer's instructions.

### Statistical analysis

All results shown are representative of at least three separate experiments. Unpaired student's *t*-test (two-tailed) or the Mann-Whitney test was used for comparison of two groups where appropriate. Kaplan Meier curve with log-rank test (GraphPad Prism 4.03 software) was used to analyze the mortality rate. *p*-value <0.05 was considered as statistically significant.

## Supporting Information

Figure S1 The location of PD-1-positive cells in the lung, kidney and heart from MHV-3 infected or PBS-treated mice was detected by immunohistochemistry (left). Statistical analysis of the number of PD-1-positive cells in the lung, kidney and heart tissue of MHV-3 infected or PBS-treated mice (right). The arrow indicates the PD-1-positive cells. Magnification ×600. NS: not significantly different.
(2.05 MB TIF)

Figure S2 (A) The architecture of the lung, kidney and heart of WT *vs.* PD-1-deficient mice after 72 h of MHV-3 infection was measured by H&E staining. (B) Cell apoptosis in these organs was detected by TUNEL staining. The arrow indicates the TUNEL-positive cells. Blue color indicates nuclear DAPI staining. Scale bar  = 20 μm. Magnification ×200.
(1.86 MB TIF)

Figure S3 The number of Foxp3-positive cells was not changed significantly in PD-1-deficient mice after MHV-3 infection. Foxp3-positive cells in the liver, thymus, spleen, and lymph nodes between PD-1-deficient and WT mice at 72 h after MHV-3 infection were detected by immunofluorescence staining (left). Statistical analysis of the number of Foxp3-positive cells in the indicated organs (right). Blue color indicates nuclear DAPI staining. Scale bar  = 20μm. NS: not significantly different.
(1.44 MB TIF)
