# Supplementary figures and images for: Programmed Death (PD)-1-Deficient Mice Are Extremely Sensitive to Murine Hepatitis Virus Strain-3 (MHV-3) Infection
Source: PLoS Pathog. 2011 Jul 7;7(7):e1001347. doi: 10.1371/journal.ppat.1001347 (PMC3131267; doi:10.1371/journal.ppat.1001347)

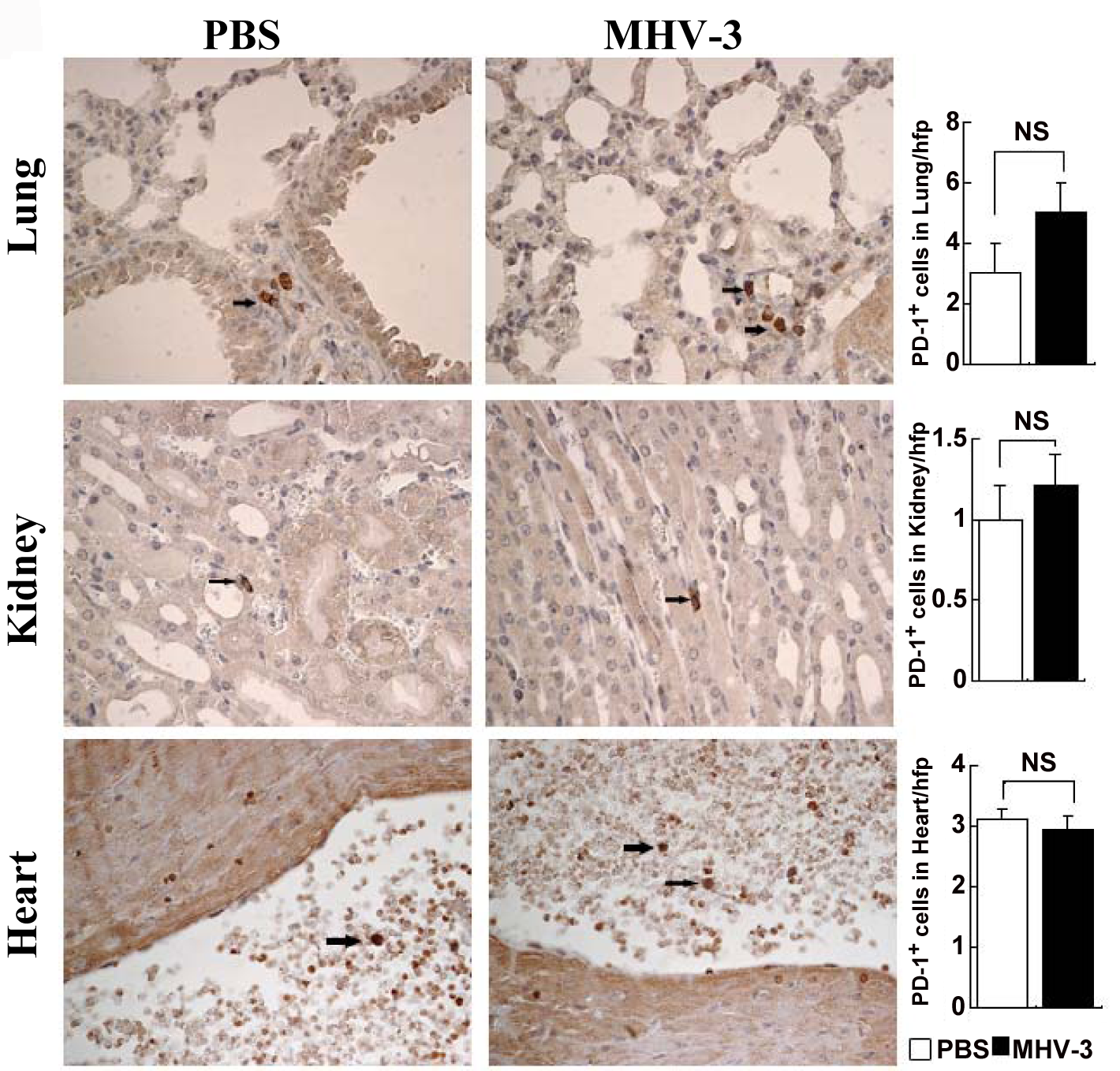

Supplement: Figure S1 — The location of PD-1-positive cells in the lung, kidney and heart from MHV-3 infected or PBS-treated mice was detected by immunohistochemistry (left). Statistical analysis of the number of PD-1-positive cells in the lung, kidney and heart tissue of MHV-3 infected or PBS-treated mice (right). The arrow indicates the PD-1-positive cells. Magnification ×600. NS: not significantly different. (2.05 MB TIF) [file ppat.1001347.s001.tif]

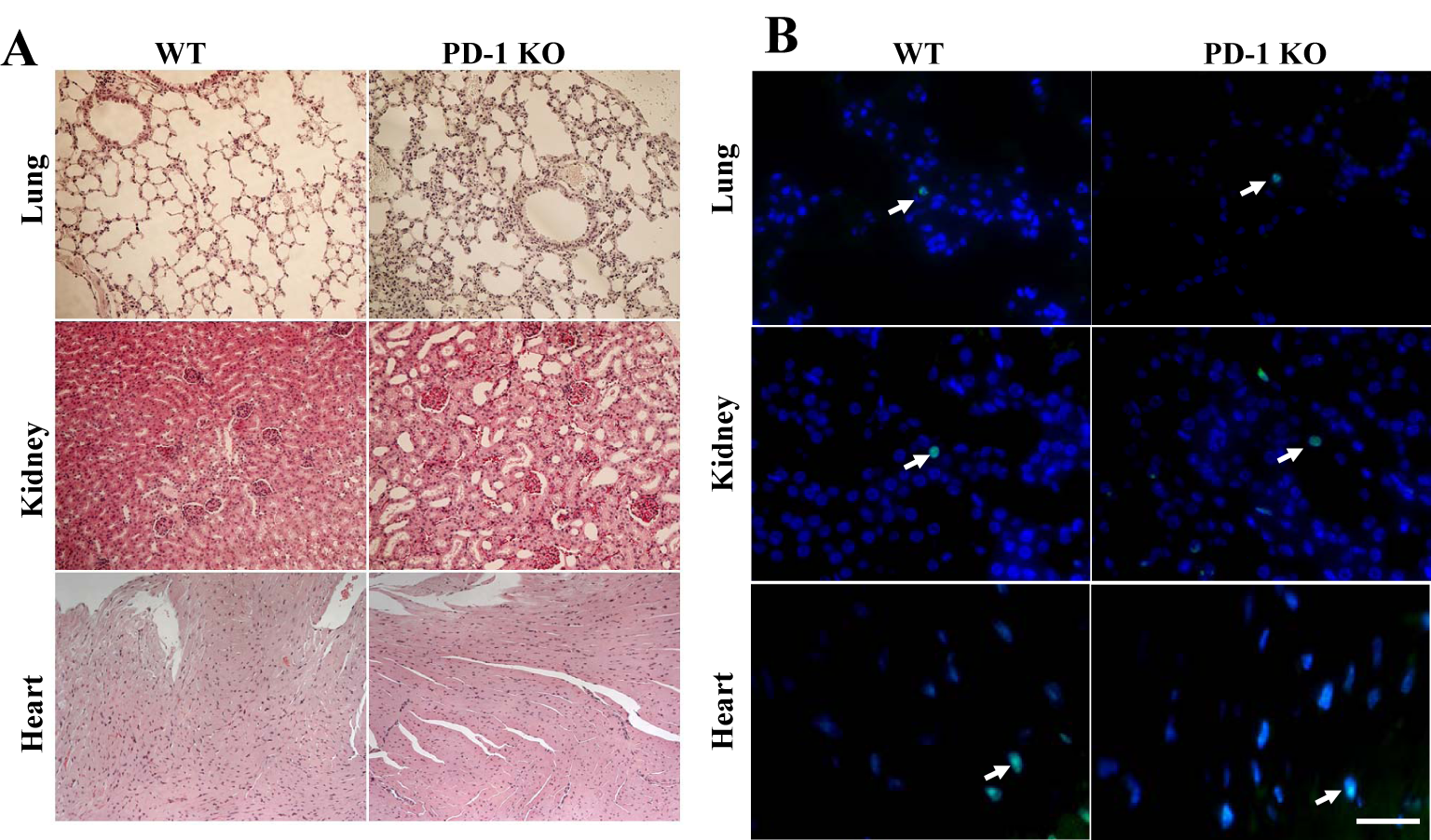

Supplement: Figure S2 — (A) The architecture of the lung, kidney and heart of WT vs. PD-1-deficient mice after 72 h of MHV-3 infection was measured by H&E staining. (B) Cell apoptosis in these organs was detected by TUNEL staining. The arrow indicates the TUNEL-positive cells. Blue color indicates nuclear DAPI staining. Scale bar = 20 μm. Magnification ×200. (1.86 MB TIF) [file ppat.1001347.s002.tif]

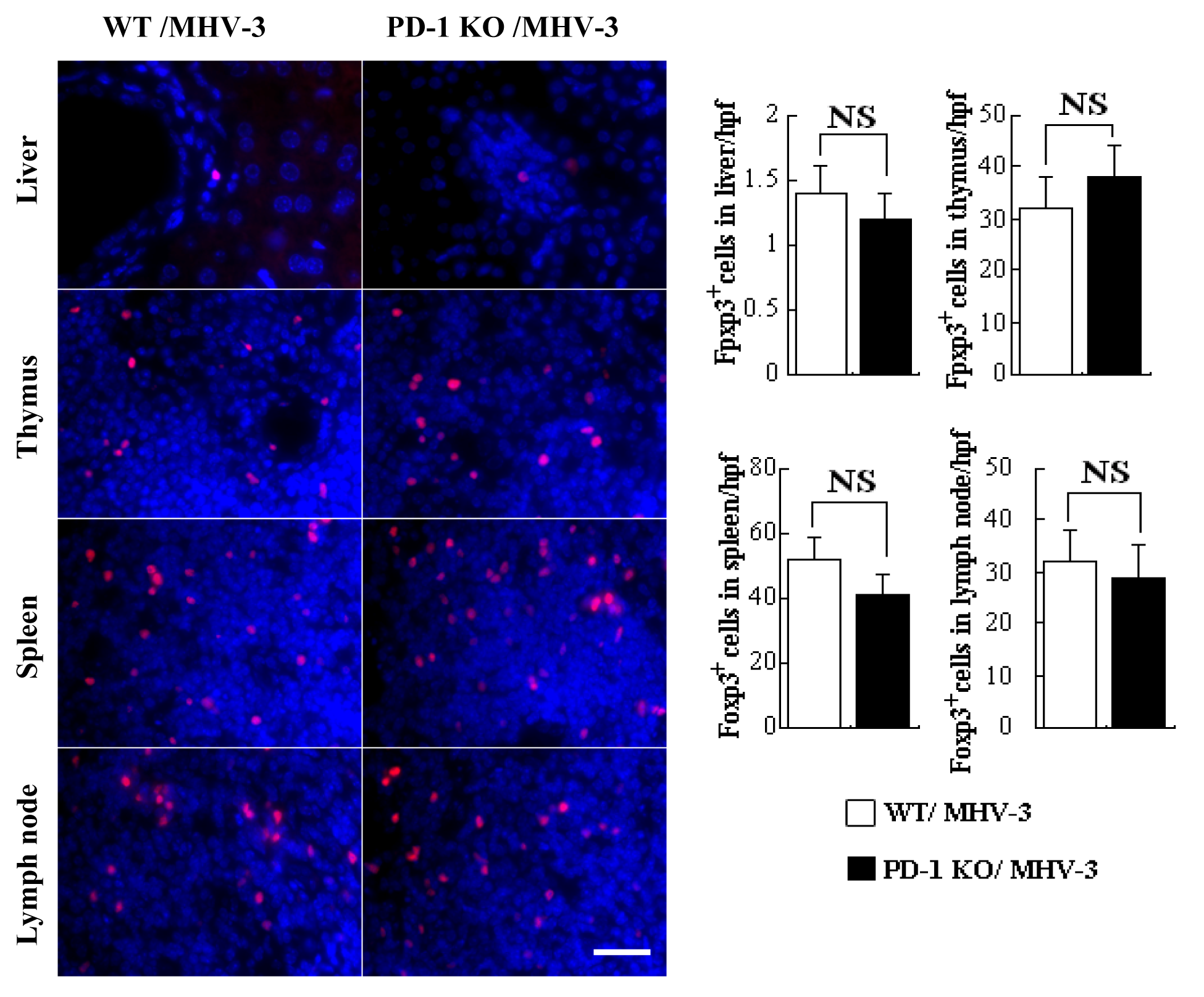

Supplement: Figure S3 — The number of Foxp3-positive cells was not changed significantly in PD-1-deficient mice after MHV-3 infection. Foxp3-positive cells in the liver, thymus, spleen, and lymph nodes between PD-1-deficient and WT mice at 72 h after MHV-3 infection were detected by immunofluorescence staining (left). Statistical analysis of the number of Foxp3-positive cells in the indicated organs (right). Blue color indicates nuclear DAPI staining. Scale bar = 20μm. NS: not significantly different. (1.44 MB TIF) [file ppat.1001347.s003.tif]
